# P171: Promoting European infection control / hospital hygiene core competencies (EIC/HHCC): a comparative analysis with related disciplines

**DOI:** 10.1186/2047-2994-2-S1-P171

**Published:** 2013-06-20

**Authors:** S Brusaferro, BD Cookson, R Gallagher, P Hartemann, J Holte, S Kalenic, W Popp, GP Privitera, CV Santos, C Suetens, L Arnoldo, G Cattani, E Fabbro

**Affiliations:** 1 Medical and Biological Sciences, University of Udine, Udine, Italy; 2 University College of London, London, UK; 3 Royal College of Nursing London, London, UK; 4 Universitè de Lorraine- CHU de Nancy, Nancy, France; 5 Staten Serum Institute Copenhagen, Copenhagen, Denmark; 6 University of Zagreb, Zagreb, Croatia; 7 University Hospital of Essen, Essen, Germany; 8 DTRM University of Pisa, Pisa, Italy; 9 ECDC Stockholm, Stockholm, Sweden; 10 Publich Health and Prevention Medicine, School of Specialization in Medical Hygiene, University of Udine, Udine, Italy; 11 Department of Biological and Medical Sciences, University of Udine, Udine, Italy

## Introduction

Training Infection Control in Europe (TRICE) in 2010 identified significant differences within European Countries (EC) in the existence of Infection Control /Hospital Hygiene (IC/HH) courses and their compliance with the Improving Patient Safety in Europe (IPSE, 2008) recommended Core Competencies. The need to improve official recognition of “IC/HH degrees” for healthcare professionals also emerged. TRICE further developed, agreed EIC/HHCC with two tiers, published by ECDC in March 2013 as a Technical Document.

## Objectives

Within the ECDC 2012 commissioned project TRICE-IS (Implementation Strategy) we conducted a comparative analysis between EIC/HHCC and IC/HH related disciplines within the broader aim of promoting mutual recognition of courses based on EIC/HHCC within European Countries.

## Methods

We collected documents about disciplines that in TRICE were related to IC/HH (Medical Microbiology, Infectious Diseases, Public Health and Epidemiology) and compared them with European IC/HH Core Competencies.

## Results

Documents collected were referred to:Public Health (ASPHER), Epidemiology (ECDC) Public Health Microbiology (EUPHEM, ECDC), Medical Microbiology (UEMS) and Infectious Diseases (UEMS). Global alignment with the EIC/HHCC competencies (n.101) resulted: 80% for ASPHER, 56% for epidemiology ECDC, 40% for Infectious Diseases, 20% Medical Microbiology UEMS and 64,4% for EUPHEM. In the Program Management area (n. 24 competencies) poor alignment has been identified for Infectious Diseases 37.5%,and Medical Microbiology (UEMS) 16.7% and Public Health Microbiology (EUPHEM) 4%.

## Conclusion

European documents addressing the training of specialties related to IC/HH have many topics similar to those reported in the EIC/HHCC. Almost all of them need to be complemented in order to cover the topics mentioned in EIC/HHCC.

## Disclosure of interest

None declared

